# Clinical Response to Baricitinib in Monogenic Lupus: Real-World Evidence from a Case Series of Seven Patients

**DOI:** 10.31138/mjr.160625.dsh

**Published:** 2026-02-04

**Authors:** Sulaiman M Al-Mayouf, Ali AlAsmari, Alhanouf Al-Saleem

**Affiliations:** 1 King Faisal Specialist Hospital & Research Centre, Riyadh, Saudi Arabia;; 2 College of Medicine, Alfaisal University, Riyadh, Saudi Arabia

**Keywords:** systemic lupus erythematosus, type I interferon, protein kinase C, complement C1q, deoxyribonucleases, baricitinib

## Abstract

**Background:**

Monogenic lupus is a rare, early-onset form of lupus caused by high-penetrance genetic variants, with diverse clinical features and a central role for type I interferon (IFN-I) in its pathogenesis. Standard treatments are lacking, but Janus kinase (JAK) inhibitors show promise, especially in refractory cases.

**Objective:**

To assess the safety and potential therapeutic benefits of baricitinib in children with monogenic lupus, based on real-world data from a single tertiary childhood lupus clinic.

**Methods:**

We conducted a retrospective observational study of patients with genetically confirmed monogenic treated with baricitinib between January 2023 and May 2025. Clinical, laboratory, and genetic data were analysed at baseline, six months, and the most recent follow-up. Outcomes included SLE Disease Activity Index (SLEDAI), Physician/Parent Global Assessment (GA), and daily glucocorticoid (GC) dose, and adverse events were collected.

**Results:**

Seven patients with mutations in *C1q, DNase1L3, PRKCD*, and *NRAS* were included. All had persistent disease activity despite multiple conventional and biologic therapies. Baricitinib was introduced as adjunct therapy. All patients tolerated baricitinib well, with no serious infections or adverse events reported. By six months, all patients showed improved disease activity, with SLEDAI scores decreasing from 14.2 to 4.1 by day 90 and remaining stable, PGA improving from 5.8 to 2.5 by day 180, and reduced daily GC doses.

**Conclusion:**

Baricitinib was well-tolerated and associated with clinical improvement in patients with treatment-refractory monogenic lupus. These findings support its potential therapeutic role and highlight the need for larger, long-term studies.

## INTRODUCTION

Systemic lupus erythematosus (SLE) is a chronic autoimmune disease characterised by heterogenous clinical manifestations and a complex interplay of genetic, hormonal, and environmental factors.^1^ In rare instances, lupus can result from high-penetrance pathogenic variants in a single gene- an entity known as monogenic lupus.^2^ This form typically presents in early childhood, with a severe clinical course marked by prominent cutaneous, neurologic, and systemic involvement, often refractory to standard treatment for SLE.^3^ Unlike classical childhood SLE (cSLE), monogenic lupus exhibits reduced female predominance and is more directly attributable to genetic defects.^4^ Genome-wide association studies have identified over 100 genomic loci associated with polygenic lupus,^5^ while more than 35 genes have been implicated in monogenic forms and lupus-like disorders.^6^

Type I interferons (type I IFNs) are critical mediators of innate immunity, particularly in response to viral infections.^7^ They are produced by various cell types and signal through the Janus kinase-signal transducer and activator of transcription (JAK-STAT) pathway, triggering the transcription of pro-inflammatory IFN-stimulated genes (ISG).^8,9^ Dysregulation of type I IFN signalling leads to interferonopathies—a group of autoinflammatory disorders that include many forms of monogenic lupus.^10^

In monogenic lupus, complement pathway deficiencies represent the most common genetic defects, while mutations in regulators of type I IFN signalling form the second most frequent category.^6^ These genetic abnormalities drive persistent ISG activation, which is often associated with severe clinical phenotypes, including renal, hematologic, and neurologic involvement.^11^ The presence of a sustained type I IFN signature has been linked to poor outcomes, providing a strong rationale for therapeutically targeting the type I IFN pathway.^12^

Many patients with monogenic SLE are not very responsive to standard treatment of SLE. Given the centrality of JAK-STAT signalling in mediating IFN responses, JAK inhibitors have emerged as promising agents for patients with monogenic lupus, particularly that refractory to conventional immunosuppressive therapies.^12–14^ In this study, we report the therapeutic effect of baricitinib in a cohort of patients with monogenic lupus, aiming to assess their clinical and laboratory response to this targeted therapy.

## MATERIALS AND METHODS

### Study design

This was a descriptive, retrospective study conducted at King Faisal Specialist Hospital and Research Centre (KFSHRC), Riyadh. The study included patients diagnosed with monogenic lupus who attended the childhood lupus clinic between January 2023 and May 2025. Eligibility criteria included: (1) a confirmed diagnosis of monogenic lupus based on genetic testing, (2) fulfilment of the 2019 EULAR/ACR classification criteria for SLE^15^, (3) diagnosis made before the age of 14, and (4) a minimum follow-up period of six months. Patients were excluded if they had insufficient clinical data.

### Data Collection

Data were extracted from electronic medical records and included demographic information, family history, clinical features, genetic findings, therapeutic interventions, and adverse events. Disease outcomes were evaluated at baseline, 3 months, 6 months, and at the most recent follow-up. Outcome measures included global disease activity assessed by the SLE Disease Activity Index (SLEDAI), Physician and Parent Global Assessments (Physician GA and Parent GA, respectively) scored on a 0 to 10 visual analog scale, and daily glucocorticoid (GC) dose.

### Safety Assessment

As a retrospective chart review, this study involved no experimental interventions. However, all patients underwent blood sampling as part of routine clinical care to monitor disease status and potential side effects. Additionally, blood samples for Polymoa BK quantitvative PCR were collected in three patients, as this was not available at our institution. All treatments were administered as part of routine clinical care, and therapeutic decisions remained at the discretion of the patients’ primary treating physicians. There were no additional risks or modifications to standard care associated with study participation.

### Ethical Considerations

This study was conducted in accordance with the ethical principles outlined in the Declaration of Helsinki (2000), the guidelines of the Research Advisory Council (RAC) of the KFSHRC, and the laws of Saudi Arabia. Ethical approval was granted by the RAC (Approval No. 2221105) on May 27, 2022. Written informed consent for genetic testing, as part of routine clinical care, was obtained from the parents of all participants. All collected data were de-identified and analysed in accordance with institutional confidentiality protocols.

## RESULTS

### Patient Cohort

Seven patients with genetically confirmed monogenic lupus were included in this study. The cohort comprised two patients with *PRKCD* deficiency, two with *C1q* deficiency, two with *DNase1L3* mutation, and one with an *NRAS* mutation. All patients demonstrated suboptimal responses to standard SLE therapies and were subsequently treated with baricitinib. It was initiated due to persistent disease activity, most notably mucocutaneous, musculoskeletal, and neurological manifestations, along with elevated inflammatory markers, and an inability to taper GC without relapse.

### Demographics and Baseline Characteristics

The median age of patients at the time of baricitinib initiation was 9 years (IQR: 7), with the median age at disease onset being 24 months (IQR: 58), and the median disease duration was 6 years (IQR: 9). Males constituted 71.4% of the cohort. All patients were of Arab origin and born to consanguineous parents.

All seven had received both GC and hydroxychloroquine (HCQ) as part of their treatment regimens. Six patients (85.7%) were treated with mycophenolate mofetil (MMF) and B-cell- targeting biologic belimumab. Intravenous immunoglobulin (IVIG) was administered in four patients (57.1%), and three patients (42.9%) also received rituximab. The detailed baseline clinical characteristics and treatment history are summarised in **Table 1**.

**Table 1.** Detailed demographic, clinical characteristics and treatment history.

**Cases**	**Gender**	**Age (Year)**	**Age at onset (Months)**	**Genetic mutation**	**Main clinical features**	**Main laboratory findings**	**Treatment**
I	M	13	2	PRKCD	BCGitis, fever, FTT, oral ulcers, lymphadenopathy, discoid lesions, arthritis, nephritis class V	Positive ANA, anti-ds-DNA, anti-Smith, anti-SSA, anti-SSB, low C3, C4, high APR	GC, HCQ, MMF, IVIG, TC, Bel, Rtx, Bari
II	M	4	2	PRKCD	Fever, BCGitis Discoid lesions, arthritis, spasticity, FTT	Positive ANA, anti-ds-DNA, anti-Smith, anti-SSA, anti-SSB, APL, low C3, C4, high APR, DCT	GC, HCQ, MMF, IVIG, Bel, Bari
III	M	9	36	DNase1L3	Arthritis, recurrent abdominal pain, diarrhoea, FTT, urticarial vasculitis, lower limb spasticity	Positive ANA, anti-ds-DNA, anti-Smith, anti-SSA, anti-SSB, APL, RF, low C3, C4, high APR	GC, HCQ, MMF, Bel, Bari
IV	M	13	108	DNase1L3	Arthritis, asthma-like, recurrent abdominal pain, urticarial vasculitis, FTT	Positive ANA, anti-ds-DNA, anti-Smith, low C3, C4, high APR	GC, HCQ, Bel, Bari
V	F	15	24	C1q	FTT, arthritis, scaring alopecia, skin ulceration, nephritis class IV	Positive ANA, anti-ds-DNA, anti-Smith, anti-SSA, anti-SSB, low C3, C4, high APR, DCT	GC, HCQ, MTX, MMF, Bel, Rtx, TC, IVIG, Bari
VI	M	8	18	C1q	FTT, arthritis, scaring alopecia, skin ulceration, lower limb spasticity	Positive ANA, anti-ds-DNA, anti-Smith, anti-SSA, anti-SSB, low C3, C4, high APR, DCT	GC, HCQ, MTX, MMF, Rtx, Bel, TC, IVIG, Bari
VII	F	6	60	NRAS	FTT, discoid lesions, asthma-like, bronchiectasis	Positive ANA, anti-ds-DNA, low C3, C4, high APR, DCT	GC, HCQ, MMF, Bari

M: Male; F: female; FTT: failure to thrive; ANA: antinuclear antibody; APL: anti-phospholipid antibody; RF: rheumatoid factor; APR: acute phase reactants; DCT: direct Coombs test; GC: glucocorticoid; HCQ: hydroxychloroquine; MMF: mycophenolate mofetil; IVIG: Intravenous immunoglobulin; TC: tacrolimus; Ble: belimumab; Rtx: rituximab; Bri: Baricitinib; MTX: methotrexate.

All patients tolerated baricitinib well, with no serious infections or adverse events reported. Haematological and biochemical parameters were routinely monitored, and no clinically significant abnormalities were observed. Additionally, all patients tested for BK virus by quantitative PCR had negative results.

### Clinical Outcomes

**Figure 1.**
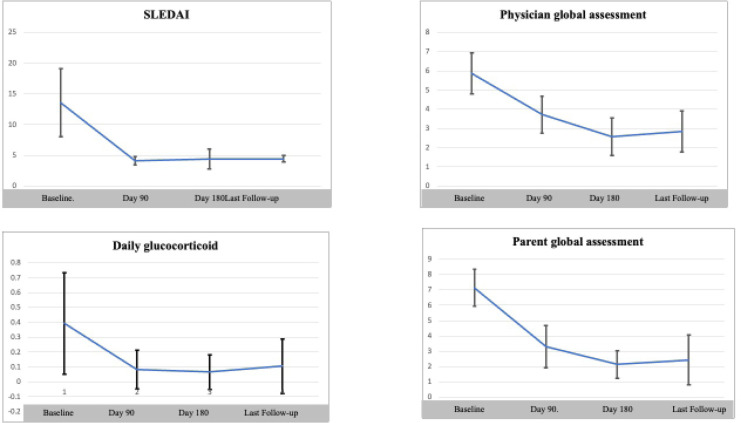
Overall clinical improvement and successful glucocorticoid tapering following baricitinib initiation.

Clinical response to baricitinib was assessed over a 180-day period. The mean SLEDAI score showed a substantial decline from 14.2 at baseline to 4.1 at 90 days and remained stable at 4.4 at 180 days and at last follow-up. Physician GA improved from a baseline of 5.8 to 3.7 in 90 days. This improvement was sustained with a mean SLEDAI of 4.4 on day 180 and the last follow-up visit. Physician GA also demonstrated progressive improvement, decreasing from a baseline score of 5.8 to 3.7 on day 90 and further to 2.5 on day 180. A slight increase to 2.8 was noted at the last follow-up, reflecting minor disease fluctuations. Similarly, the Parent GA showed marked improvement, decreasing from 7.1 at baseline to 3.2 at 90 days and 2.1 at 180 days, remaining at 2.4 at last follow-up. The mean daily GC dose declined from 0.4 mg/kg/day at baseline to 0.08 mg/kg/day at 90 days, further tapering to 0.06 mg/kg/day at 180 days. At last follow-up, the mean GC dose remained low at 0.1 mg/kg/day. **Figure 1** shows overall clinical improvement and successful glucocorticoid tapering following baricitinib initiation.

Despite notable clinical improvements, changes in laboratory biomarkers were limited. Complement C3, C4 levels, anti-dsDNA, and urine protein-to-creatinine ratio exhibited minimal or inconsistent improvement, particularly in patients with baseline hypocomplementemia. Detailed data are presented in **Table 2**.

**Table 2.** The relationship between some clinical conditions and KIM-1 in the FMF group.

**Cases**	**Outcome measure**	**Baseline**	**90 Days**	**180 Days**	**Last follow-up visit**
I	SLEDAI	25	4	8	5
	UPCR	143	44	32	
	C3 g/L (0.9–1.8)	0.65	0.48	0.88	
	C4 g/L (0.1–0.4)	0.04	0.04	0.07	
	Anti dsDNA (<200)	553	172	118	
	Physician GA	7	3	3	3
	Parent GA	8	2	3	2
	GC dose (mg/kg/day)	0.66	0.35	0.20	0.09
II	SLEDAI	10	4	4	4
	UPCR	100	-	-	
	C3 g/L (0.9–1.8)	0.65	0.70	0.88	
	C4 g/L (0.1–0.4)	0.06	0.06	0.07	
	Anti dsDNA (<200)	419	443	-	
	Physician GA	6	4	3	4
	Parent GA	5	2	2	2
	GC dose (mg/kg/day)	0.44	Off	Off	Off
III	SLEDAI	10	3	3	5
	UPCR	28	22	33	
	C3 g/L (0.9–1.8)	0.56	0.57	0.60	
	C4 g/L (0.1–0.4)	0.09	0.12	0.11	
	Anti dsDNA (<200)	24	22	-	
	Physician GA	4	3	1	4
	Parent GA	7	5	1	6
	GC dose (mg/kg/day)	Off	Off	Off	0.5
IV	SLEDAI	12	5	4	4
	UPCR	21	-	12	
	C3 g/L (0.9–1.8)	0.69	0.61	0.63	
	C4 g/L (0.1–0.4)	0.09	0.06	0.05	
	Anti dsDNA (<200)	19	-	24	
	Physician GA	6	3	2	1
	Parent GA	8	2	1	1
	GC dose (mg/kg/day)	1	Off	Off	Off
V	SLEDAI	14	4	4	4
	UPCR	22	31	28	
	C3 g/L (0.9–1.8)	2.29	1.87	1.62	
	C4 g/L (0.1–0.4)	0.42	0.28	0.25	
	Anti dsDNA (<200)	32	27	25	
	Physician GA	7	5	4	3
	Parent GA	8	5	2	2
	GC dose (mg/kg/day)	0.2	0.1	Off	Off
VI	SLEDAI	15	5	4	5
	UPCR	36	35	29	
	C3 g/L (0.9–1.8)	1.56	1.47	1.43	
	C4 g/L (0.1-0.4)	0.31	0.21	0.22	
	Anti dsDNA (<200)	49	21	33	
	Physician GA	6	5	3	3
	Parent GA	8	4	3	2
	GC dose (mg/kg/day)	30 then 0.15	0.13	Off	Off
VII	SLEDAI	9	4	4	4
	UPCR	16	17	19	
	C3 g/L (0.9–1.8)	1.45	-	1.16	
	C4 g/L (0.1–0.4)	0.47	-	0.53	
	Anti dsDNA (<200)	343	-	-	
	Physician GA	5	3	2	2
	Parent GA	6	3	3	2
	GC dose (mg/kg/day)	0.3	Off	0.27	0.15

SLEDAI: Systemic lupus erythematosus disease activity index; UPCR: urine protein-creatinine ratio; GA: global assessment; GC: glucocorticoid.

## DISCUSSION

This study highlights the potential role of baricitinib as an adjunctive therapy in children with genetically confirmed monogenic lupus. Our cohort, though small, reflects the clinical heterogeneity and treatment-refractory nature characteristic of this rare form of lupus.

The observed improvements in global disease activity scores (SLEDAI, physician, and parent GA) and reduction in GC dosage over at least a 6-month period underscore the potential of targeting the IFN-I pathway in these patients.

Notably, all patients had previously exhibited suboptimal responses to conventional SLE therapies, including biologic agents such as belimumab and rituximab. The decision to initiate baricitinib was largely driven by persistent disease activity, particularly mucocutaneous, musculoskeletal, and neurologic symptoms—domains where interferon signalling has been implicated as a central pathogenic driver. The consistent decline in SLEDAI scores, along with marked improvements in patient and physician global assessments, supports the hypothesis that JAK inhibition may disrupt this pathogenic feedback loop, particularly in IFN-driven disease.^16^ Notably, GC burden was significantly reduced during baricitinib therapy, supporting a steroid-sparing effect of baricitinib.

Interestingly, laboratory markers such as complement levels and anti-dsDNA titres remained largely unchanged in most patients, suggesting a clinical-laboratory dissociation. This finding may reflect the limitations of conventional serologic markers in capturing interferon-mediated disease activity, or the complex interplay between innate immune activation and serologic autoimmunity in monogenic lupus. Similar discrepancies have been reported in interferonopathies, where clinical improvements precede or occur independently of serologic normalisation.^17^

The demographic characteristics of our cohort - early disease onset, high male representation, consanguinity, and Arab ethnicity - are consistent with previously published data on monogenic lupus. The genetic heterogeneity of the cohort (mutations in *PRKCD*, *C1q*, *DNase1L3*, and *NRAS*) also reflects the diverse molecular pathways converging on a shared lupus phenotype, further underscoring the need for pathway-specific therapies such as JAK inhibitors.^18^

The availability of guidelines for the treatment of childhood SLE provides the foundation of standard care for treatment. However, the treatment of monogenic lupus lacks such guidelines, which represents a challenge. JAK inhibitor therapy provides clinical benefits for patients with SLE, including children.^19^

In 12 patients with genetically confirmed C1q deficiency and SLE-like manifestations, three patients were treated with baricitinib, with variable outcomes; one displayed an apparently favourable response with respect to cutaneous and neurological features, and two others experienced persistent disease despite baricitinib.^20^

Our study’s main strength, while a recent report has described the use of baricitinib in monogenic lupus, our study builds upon this by presenting a series of genetically confirmed, treatment-refractory cases with heterogeneous clinical phenotypes.^21^ It offers novel therapeutic insights and real-world experience with JAK inhibition in rare and challenging patient population, thereby expanding the current understanding of targeted treatment strategies in monogenic lupus. Despite encouraging findings, our study has limitations. The retrospective, single-centre design and small sample size limit generalisability. In addition, while short-term safety and clinical efficacy were favourable, longer follow-up is needed to assess sustained response, relapse rates, and long-term adverse effects of JAK inhibition in paediatric populations. Moreover, the absence of a control group prevents firm conclusions regarding causality, though the temporal relationship between baricitinib initiation and clinical improvement is notable.

## CONCLUSION

This study presents baricitinib as a potential adjunctive therapy for children with monogenic lupus. Future prospective studies should explore the role of baricitinib in combination with other targeted agents, as well as biomarkers that better predict treatment response in monogenic lupus. Additionally, deeper immunopheno-typing and transcriptomic profiling could help clarify the mechanisms underlying differential treatment responses and refine patient selection for JAK inhibitor therapy.
